# Casein Kinase 1δ/ε Inhibitor, PF670462 Attenuates the Fibrogenic Effects of Transforming Growth Factor-β in Pulmonary Fibrosis

**DOI:** 10.3389/fphar.2018.00738

**Published:** 2018-07-10

**Authors:** Christine R. Keenan, Shenna Y. Langenbach, Fernando Jativa, Trudi Harris, Meina Li, Qianyu Chen, Yuxiu Xia, Bryan Gao, Michael J. Schuliga, Jade Jaffar, Danica Prodanovic, Yan Tu, Asres Berhan, Peter V. S. Lee, Glen P. Westall, Alastair G. Stewart

**Affiliations:** ^1^Lung Health Research Centre, Department of Pharmacology and Therapeutics, University of Melbourne, Parkville, VIC, Australia; ^2^Department of Biomedical Engineering, University of Melbourne, Parkville, VIC, Australia; ^3^ARC Centre for Personalised Therapeutics Technologies, Parkville, VIC, Australia; ^4^Department of Allergy, Immunology and Respiratory Medicine, The Alfred Hospital, Monash University, Melbourne, VIC, Australia

**Keywords:** PF-670462, TGF-β, lung, mechanobiology, collagen, epithelial mesenchymal transition, myofibroblast, anti-fibrotic

## Abstract

Transforming growth factor-beta (TGF-β) is a major mediator of fibrotic diseases, including idiopathic pulmonary fibrosis (IPF). However, therapeutic global inhibition of TGF-β is limited by unwanted immunosuppression and mitral valve defects. We performed an extensive literature search to uncover a little-known connection between TGF-β signaling and casein kinase (CK) activity. We have examined the abundance of CK1 delta and epsilon (CK1δ/ε) in lung tissue from IPF patients and non-diseased controls, and investigated whether inhibition of CK1δ/ε with PF670462 inhibits pulmonary fibrosis. CK1δ/ε levels in lung tissue from IPF patients and non-diseased controls were assessed by immunohistochemistry. Anti-fibrotic effects of the CK1δ/ε inhibitor PF670462 were assessed in pre-clinical models, including acute and chronic bleomycin mouse models and *in vitro* experiments on spheroids made from primary human lung fibroblast cells from IPF and control donors, and human A549 alveolar-like adenocarcinoma-derived epithelial cells. Increased expression of CK1δ and ε in IPF lungs compared to non-diseased controls was accompanied by increased levels of the product, phospho-period 2. *In vitro*, PF670462 prevented TGF-β-induced epithelial-mesenchymal transition. The stiffness of IPF-derived spheroids was reduced by PF670462 and TGF-β-induced fibrogenic gene expression was inhibited. The CK1δ/ε inhibitor PF670462 administered systemically or locally by inhalation prevented both acute and chronic bleomycin-induced pulmonary fibrosis in mice. PF670462 administered in a ‘therapeutic’ regimen (day 7 onward) prevented bleomycin-induced lung collagen accumulation. Elevated expression and activity of CK1 δ and ε in IPF and anti-fibrogenic effects of the dual CK1δ/ε inhibitor, PF670462, support CK1δ/ε as novel therapeutic targets for IPF.

## Introduction

Fibrosis, a common feature of most chronic diseases, contributes to up to 45% of deaths in the industrialized world (Friedman et al., 2013). Idiopathic pulmonary fibrosis (IPF) is an irreversible, progressive and usually fatal lung disease characterized by fibrosis of the lung parenchyma and progressive loss of lung function, with a median survival following diagnosis of 2.5–3.5 years (Ley et al., 2011). Drug targeting of IPF is hampered by a lack of understanding of its pathogenesis (Spagnolo et al., 2014). Current evidence suggests that dysregulated repair of injured alveolar epithelial cells leads to the subepithelial accumulation of activated myofibroblasts through the proliferation and migration of interstitial fibroblasts, epithelial-mesenchymal transition (EMT), and recruitment of circulating fibrocytes, leading to fibroplasia and excessive deposition of collagen within the lung interstitium and alveolar space (Fernandez and Eickelberg, 2012). The proliferation and migration of mesothelial cells and pericytes also contribute to the development of fibroblast foci that form a complex, three-dimensional reticulum from the pleural surface into the lung parenchyma. The quantitative contribution of these respective sources of activated fibroblasts to the overall fibroplasia is controversial (see reviews: Kage and Borok, 2012; Bartis and Thickett, 2014; Bartis et al., 2014; Wolters et al., 2014; Mora et al., 2017), but unresolved in this condition which remains idiopathic. Patients experience dyspnea and cough due to progressive fibrosis and stiffening of the lungs. The mechanical changes lead to increased lung elasticity, restrictive ventilation impairment, and ultimately to respiratory failure (Raghu et al., 2011).

Transforming growth factor-beta (TGF-β) makes a major contribution to fibrotic disease through, *inter alia*, induction of EMT and activation of myofibroblasts (Leask and Abraham, 2004). The 3 isoforms of TGF-β (TGF-β_1-3_) share similar biological activity, but differ in expression patterns. TGF-β_1_ is the only isoform shown to be differentially expressed in epithelial cells from advanced pulmonary fibrosis (Khalil et al., 1996). Transient pulmonary overexpression of TGF-β_1_ is sufficient to phenocopy progressive lung fibrosis in mice (Sime et al., 1997; Coker et al., 2001). TGF-β_1_ and associated down-stream signaling pathways therefore present as prominent therapeutic targets for the treatment of pulmonary fibrosis.

Induction of fibrosis by TGF-β_1_ is mediated by ALK5-dependent pathways (Biernacka et al., 2011). However, global inhibition of TGF-β_1_ signaling through the inhibition of ALK5 receptor kinase activity is not a feasible therapeutic approach, as it results in autoimmune colitis (Shull et al., 1992; Kulkarni et al., 1993) and mitral valve damage (Anderton et al., 2011). In contrast, the modulators of TGF-β_1_ expression and activity, nintedanib and pirfenidone, are sufficiently well-tolerated for their chronic use in IPF. Thus, partial abrogation of TGF-β_1_ activity presents a clinically tractable approach to the treatment of fibrosis (LaChapelle et al., 2018). Our efforts to identify the mechanism of TGF-β-induced glucocorticoid insensitivity (Salem et al., 2012; Keenan et al., 2014; Xia et al., 2017) lead to a comprehensive literature review that revealed a little-known connection between TGF-β_1_ signaling and casein kinase (CK) activity (Hall et al., 1996). CK1ε physically interacts with Smad3 to mediate some TGF-β signals, including upregulation of the fibrogen, plasminogen-activator inhibitor-1 (Waddell et al., 2004). Since CK1ε shares much functional redundancy with CK1δ due to 98% homology in the kinase domains (Fish et al., 1995), in the current study, we sought to investigate whether CK1δ and/or ε are dysregulated in the lungs of patients with IPF. Furthermore, we investigate whether PF670462, a dual inhibitor of casein kinase 1δ/ε activity with Ki values of 10 and 50 nM respectively (Badura et al., 2007; Wager et al., 2014), has anti-fibrotic effects in pre-clinical models of pulmonary fibrosis. This dual CK1δ/ε inhibitor has effects on the circadian clock by blocking the phosphorylation of the CLOCK (circadian locomotor output cycles kaput) repressor, Period (Badura et al., 2007; Wager et al., 2014). The Period protein (Per-2) acts to repress CLOCK genes, including ARNTL which encodes the transcription factor, BMal1 (Dierickx et al., 2018). The phosphorylation of Per-2 by CK1δ inactivates its transrepressive activity. Conversely, inhibition of CK1δ leads to an initial increase in Per-2 with more repression of ARNTL. We have therefore used ARNTL expression in the current study to establish an effective blockade of CK1δ. Further interest in our study is provided by findings implicating CLOCK and related CLOCK-dependent genes in airway inflammation and fibrosis (Durrington et al., 2014). In order to avoid the confounding influence of central CLOCK disruption, we have established the anti-fibrogenic effectiveness of inhaled PF670462, demonstrating the feasibility of lung-selective therapy.

## Materials and Methods

### Cell Culture

Primary human parenchymal fibroblast cells (pFbs) were cultured from parenchyma of lung resection specimens from non-transplanted lungs of donors without chronic respiratory disease and those of IPF patients diagnosed by multidisciplinary review (HREC #336/13; Alfred Hospital, Melbourne VIC, Australia), as previously described (Schuliga et al., 2009, 2017). Donor characteristics of specimens used for cell culture are shown in **Table 1**. pFbs were passaged in Dulbercco’s Modified Eagle’s Media (DMEM) containing 10% (v/v) heat-inactivated fetal calf serum (FCS), 15 mM HEPES, 0.2% (v/v) sodium bicarbonate, 2 mM L-glutamine, 1% (v/v) non-essential amino acids, 1% (v/v) sodium pyruvate, 2.5 μg/mL amphotericin, 5 IU/mL penicillin and 50 μg/mL streptomycin. Primary fibroblast 3D spheroids were generated by seeding cells into 96 well round bottom plates coated with 0.5% poly(2-hydroxyethyl methacrylate) (poly-HEMA) to minimize cellular adhesion to plastic, and by incubating cells to allow aggregation into cellular spheroids over a period of 24–48 h.

**Table 1:** Characteristics of lung tissue obtained from IPF and non-IPF donors for CK1δ/ε immunohistochemistry.

Age	Sex	FEV1 (% pred)	FVC (% pred)	TLCO (% pred)	Smoking history (pack yrs)
**Control patients**					
47.4 ± 16.7 (mean ± SD)	2M/2F/2 unknown	*unknown*	*unknown*	*unknown*	4Ex/1 current/1 unknown
**IPF patients (diagnosed by interdisciplinary review**)					
60.5 ± 4.6 (mean ± SD)	4M/2F	62.3 ± 13.6 (mean ± SD)	53.3 ± 16.4 (mean ± SD)	14.8 ± 1.6 (mean ± SD)	4 current/2 never

A549 alveolar epithelial-like adenocarcinoma-derived cells (ATCC, Manasas, VA, United States) were cultured in DMEM containing 5% (v/v) FCS, 15 mM HEPES, 0.2% (v/v) sodium bicarbonate, 2 mM L-glutamine, 1% (v/v) non-essential amino acids, 1% (v/v) sodium pyruvate, 5 IU/mL penicillin and 50 μg/mL streptomycin as previously described (Salem et al., 2012; Keenan et al., 2014).

Prior to experimentation, pFb and A549 cells were incubated in serum-free DMEM containing 0.25% bovine serum albumin (BSA) and insulin-transferrin-selenium–containing supplement (Monomed A; CSL, Parkville, Melbourne, VIC, Australia). Where indicated, cells were treated with PF670462 (0.3 – 10 μM) (Abcam, Australia) prior to 100 pM TGF-β_1_ (R&D Systems, Minneapolis, MN, United States) or 300 pM bFGF (Promega, Madison, WI, United States). Pirfenidone, PF670462 (Abcam, Australia), and nintedanib (Focus Bioscience, Australia) were made as 10 mM – 100 mM stock solutions in 100% DMSO and diluted to the required concentration in medium or saline containing 0.1% DMSO (final concentration). For intraperitoneal administration of PF670462 it was initially dissolved in 100% DMSO and prepared for injection by 1:10 dilution in Arachis oil (Sigma). For inhalational studies PF670462 was dissolved in the indicated concentrations in saline for injection.

### Cell Stiffness Measurement

The micropipette aspiration technique was used to measure the Young’s tensile modulus (*E*) of 3D spheroids, as previously described (Guevorkian et al., 2010, #39; Schuliga et al., 2013, #38). The stiffness of individual fibroblast cells was also assessed following dissociation from plastic culture vessels using trypsin. The micropipette aspiration was performed in a temperature control chamber (Warner Instrument CL-100) to maintain the cells at 37°C. Briefly, the tips of the custom-made glass micropipettes (8 μm diameter), were pre-coated with silicone using Sigmacote (Sigma, United Kingdom) to prevent cell adhesion. Suction pressure was applied to the cell/cell aggregate by controlling the water level in a water reservoir. The suction pressure was applied in 1 cm H_2_O (0.098 kPa) increments and kept stable for 60 s at each increment. The maximum suction pressure was 6 cm H_2_O (0.588 kPa). The aspirated length in the micropipette was measured from a brightfield image taken by a Leica DMI6000B microscope at the end of each pressure increment. The apparent Young’s modulus of the cell (or cell stiffness) was calculated using a model that assumes the cell is a homogeneous, isotropic, elastic and incompressible half-space medium (Theret et al., 1988, #40). Young’s Modulus *E* was calculated according to Equation 1 where *Dp* is the inner diameter of the micropipette and *L* the aspiration length, as shown in **Figure 3**. Δ*P* represents the aspiration pressure and *ϕ* is the wall function with a typical value of 2.1, as described previously (Hochmuth, 2000).

(1) $$E=\frac{3Dp\Delta P}{4\pi L\phi }$$

### Immunohistochemistry

CK1δ and ε expression was assessed by immunohistochemistry (IHC) in lung tissue specimens from IPF patients and non-diseased controls. Sections were obtained from end stage IPF patients undergoing lung transplantation and from controls without IPF. Donor characteristics for the IHC studies are provided in **Table 2**.

**Table 2:** Donor characteristics for human primary fibroblast cultures.

Age	Sex	FEV1 (% pred)	FVC (% pred)	TLCO (% pred)	Smoking history (pack yrs)
**Control patients**					
50.2 ± 14.6 (mean ± SD)	7M/5F/1 unknown	*unknown*	*unknown*	*unknown*	*7 unknown; 5 ex; 1 current*
**IPF patients (diagnosed by interdisciplinary review)**					
58.0 ± 8.2 (mean ± SD)	7M/1F	53.8 ± 16.2 (mean ± SD)	48.7 ± 18.6 (mean ± SD)	22.0 ± 9.4 (mean ± SD)	2 Never/4 ex/2 unknown

Paraffin-embedded IPF and non IPF lung tissue was obtained from Alfred Health from the Alfred Tissue Biobank for Interstitial Lung Disease (HREC#336/13, Alfred Hospital, Melbourne, VIC, Australia). Sections of parenchymal lung tissue were immunostained for one of CK1δ (rabbit, 1:400, Abcam, Cambridge, United Kingdom), CK1ε (rabbit, 1:25, Proteintech Group, Chicago, IL, United States) or positive control pan-actin (rabbit, 1:400, Cell Signaling, Danvers, MA, United States). Immunohistochemistry was completed by using the Vectastain ABC kit (Biotinylated goat anti rabbit, 1:200, streptavidin HRP layer 1:100, Vectalabs, United States) and 3, 3-diaminobenzidine development for visualization (Dako chromagen substrate kit, United States). Sections were counterstained with haematoxylin (Grale Scientific, Australia). On serial sections, a Masson’s Trichrome stain was also performed to provide indicative levels of fibrosis in IPF and non-IPF sections the reagents required include Bouin’s fixative solution Scott’s tap water, Mayer’s Haematoxylin, Trajan Scientific; Celestine Blue, Biebrich Scarlet/Acid Fuchsin; Phosphotungstic acid, Sigma; Aniline blue solution (aust biostain)]. Selected sections were also stained with anti-α-smooth muscle actin (mouse monoclonal 1:400, Dako Cytomation). The CK1δ and CK1ε staining was qualitatively scored from 0 to 5 by an experienced operator and confirmed by an additional operator (each blinded to group allocations), with 0 denoting no staining and 5 indicating heavy uniform and extensive staining.

### Immunofluorescence

A549 cells for immunofluorescence staining were seeded in ibiTreat 8-chamber slides (Ibidi) and left to adhere overnight. Cells were then serum-starved for 16 h prior to pre-incubation with PF670462 (0.3 – 10 μM) for 30 min then TGF-β (100 pM) for 48 h. Cells were fixed in 10% neutral buffered formalin (Grale Scientific) for 15 min and non-specific binding sites were blocked by incubation with 5% normal goat serum/0.3% Triton X-100 in PBS for 1 h. *E*-Cadherin expression was detected using a rabbit monoclonal antibody (1:200, Clone 24E10; Cat#3195, Cell Signaling) followed by an AlexaFluor-488 conjugated anti-Rabbit F(ab′)_2_ fragment secondary (1:500, Cat#4412, Cell Signaling). Specific binding was confirmed using an isotype control antibody (protein content matched to respective primary antibodies, Clone DA1E rabbit IgG; Cat#3900, Cell Signaling). Cell nuclei were then stained with DAPI. Cells were imaged using a Leica SP5 confocal microscope (Biological Optical Microscopy Platform, University of Melbourne). Cell morphology and immunofluorescent staining was quantified using the Operetta High Content Imaging System (Biological Optical Microscopy Platform, University of Melbourne).

### Bleomycin-Model of Pulmonary Fibrosis

All animal experiments were carried out in accordance with ethical guidelines from the University of Melbourne Animal Ethics Committee (AEEC#1513736.1). Six- to eight-week old 20–25 g C57Bl/6 mice (ARC, Perth, WA, Australia) received 35 μL of saline or bleomycin (105 mU per mouse) on day 0 by intranasal administration, as previously described (Langenbach et al., 2007). Acute and chronic bleomycin mouse models were used to assess the effect of PF670462 on pulmonary fibrosis *in vivo*. PF670462 was administered systemically by intraperitoneal injection or locally to the lungs by inhalation of an aerosol generated using an oxygen concentrator connected to a Hudson nebuliser operating at 5 L/min for 15 min. Mice that did not receive PF670462 treatment received vehicle (intraperitoneal administration,10% DMSO, 90% peanut oil; inhalational administration, normal saline). In the 3-day model, PF670462 administration commenced on day −1 (day prior to bleomycin) and continued to day 3. In the 14-day model PF670462 was administered from day 3 to day 14. In the 21-day model considered to reflect the peak of fibrosis (Langenbach et al., 2007), PF670462 administration commenced on day 8 and continued to day 21. Mice were allowed food and water *ad libitum* for the duration of all studies. At the end of each study, bronchoalveolar lavage (BAL) was performed from which viable cells were enumerated (cells that show no nuclear staining after incubation on ice for 30 s in acridine orange/ethidium bromide/saline resuspension of the cell pellet sedimented by centrifugation at 1000 × *g*, 5 min 4°C) with the aid of an epifluorescence microscope (Zeiss Axioshop, Germany). Acellular protein content in the supernatant of the sedimented BAL cell pellet was determined by the Bradford protein assay using BSA as a standard, as previously described (Langenbach et al., 2007), lungs were dissected and snap frozen. Hydroxyproline content, phosphorylated p38 MAP kinase levels and gene expression were determined from pulverized frozen lung tissue, as previously described (Langenbach et al., 2007; Salem et al., 2012).

### Lung Dry Mass and Hydroxyproline Determination

Frozen mouse lung tissue was pulverized in a mortar and pestle chilled by liquid N2. The lung fragments were weighed and then lyophilized and re-weighed to determine dry mass as previously described (Langenbach et al., 2007). Briefly, hydroxyproline content was then determined from 8 mg of lyophilized mouse lung tissue by hydrolysis in 6M HCL for 16 h at 130°C. Ten microliters of the hydrolysate was added to a well of a 96 well plate containing 10 μL of citrate buffer, to which 100 μL of the chromagen chloramine T (Sigma–Aldrich, United States) was added for 20 min at ambient temperature prior to the addition of 100 μM 4-(dimethylamino)benzaldehyde (DMAB, Fluka, Switzerland). The absorbance was measured at 560 nm in a multiscan plate reader after being allowed to cool for 10 min following 20 min incubation at 65°C, with further information as previously detailed (Langenbach et al., 2007).

### Protein Analysis and Immunoassay

Total protein content in acellular BAL fluid, generated by centrifugation at 1000 × *g*, 5 min 4°C, was assessed using the Bradford protein assay method (BioRad, Australia) with BSA as a standard. Levels of phosphorylated p38 MAP kinase were determined in lyophilized mouse lung tissue by western blotting, as previously described (Salem et al., 2012). A limited number of BAL samples from the 3-day systemic treatment study were submitted to immunoassay for IL-6, which was carried out according to the Manufacturer’s instructions (murine OptiEIA kit, Becton Dickinson, Australia).

### Analysis of Gene Expression

Total RNA was extracted from pulverized mouse lung or cultured cells using illustra RNAspin Mini RNA Isolation Kit (GE Healthcare). RNA extracts were reverse transcribed into cDNA using High-Capacity RNA-to-cDNA Kit (Applied Biosystems). Real-time PCR was then performed using a QuantStudio 6 Flex Real-Time PCR System using iTaq Universal SYBR green supermix and the following thermal protocol: 50°C (2 min), 95°C (10 min), then 40 cycles of 95°C (15 s), 60°C (1 min). The threshold cycle (CT) values determined for target genes were normalized against those obtained for 18S ribosomal RNA (18S rRNA), which was included as internal control. The generation of specific PCR products was confirmed by dissociation curve analysis. Primer sequences used are shown in **Table 3**.

**Table 3:** Primer sequences for RT-qPCR.

Gene	Forward primer	Reverse primer
**Human**		
18S rRNA	CGC CGC TAG AGG TGA AAT TC	TTG GCA AAT GCT TTC GCT C
COL1A	GTG CTA AAG GTG CCA ATG GT	ACC AGG TTC ACC GCT GTT AC
CTGF	TGT GTG ACG AGC CCA AGG A	TCT GGG CCA AAC GTG TCT TC
E-Cad	ACC ACA AAT CCA GTG AAC AAC G	CAA GCC CTT TGC TGT TTT CAA
*N*-Cad	CGA GAA AAA GTG CAA CAG TAT ACG TTA A	GCC TTC CAT GTC TGT AGC TTG A
PAI-1	TCAGGCTGACTTCACGAGTCTTT	CTGCGCGACGTGGAGAG
VIM	AAT CCA AGT TTG CTG ACC TCT CTG	GGG CGT CAT TGT TCC GG
**Mouse**		
18s rRNA	TCC GGC GAG GGA GCC TG	CCT GCT GCC TTC CTT GGA T
ARG2	CAT AAT ACA GGG TTG CTG TC	CTT CTC TTG TCT GAC CAA AAC
COL1A	ACG GCT GCA CGA GTC ACA C	GGC AGG CGG GAG GTC TT
COL3	GTT CTA GAG GAT GGC TGT ACT AAA CAC A	TTG CCT TGC GTG TTT GAT ATT C
CTGF	GTC AAG CTG CCT GGG AAA TG	CTT GGG CTC GTC ACA CAC C
IFN-λ2	AAG GAT GCC ATC GAG AAG	GTC ATG TTC TCC CAG ACC
IL-6	CTG CAA GAG ACT TCC ATC CAG TT	TTG TCA CCA GCA TCA GTC CC
MMP-2	ATC ATT GGT TAC ACA CCT GAC CTG	GCA AAA GCA TCA TCC ACG G
PAI-1	AGC AAC AAG TTC AAC TAC AC	CTT CCA TTG TCT GAT GAG TTC
TIMP-1	GAT ATG CCC ACA AGT CCC AGA	GGC CCG TGA TGA GAA ACT CTT
TIMP-2	GAC GTA GTG ATC AGA GCC AAA GC	CCC GGA ATC CAC CTC CTT

### Statistical Analysis

**FIGURE 1:**
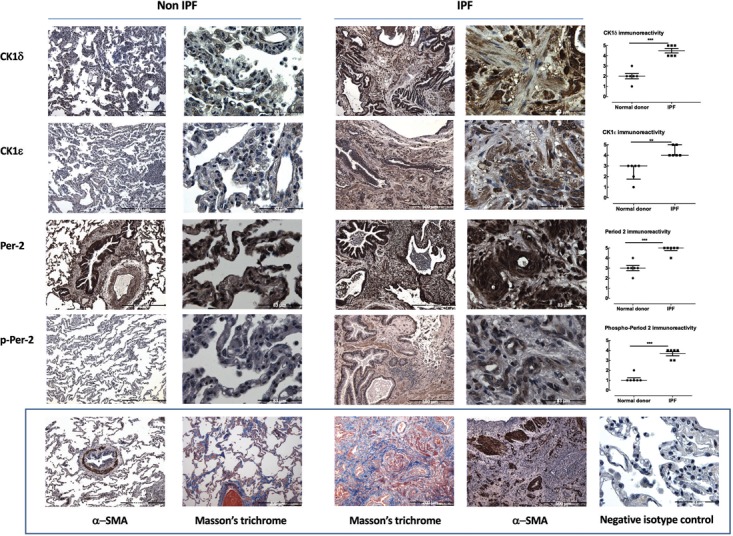
CK1δ and ε and their product phosphorylated period-2 are highly upregulated in the lungs of IPF patients. CK1δ, CK1ε, Period-2 and phospho-period 2 levels are greater in IPF (*n* = 6) compared to non-IPF donor lung (*n* = 6) parenchyma (representative IPF and non-IPF sections are accompanied by blinded assessments of staining (on a scale of 0–5) and tested by Mann–Whitney *U*-test. The bottom panel shows representative Non-IPF and IPF α-smooth muscle actin, a non-IPF isotype IgG control and Masson’s Trichrome stain in both IPF and non-IPF lung parenchyma. These latter images are not intended for quantitative analysis but to indicate the level of fibrosis, positive and negative immunostaining to contextualize the images in the rows above. Scale bars identify images with different levels of magnification.

Data are presented as mean ± SEM or SD (**Tables 1**, **2**), as indicated. Statistical comparisons among multiple groups were made by 1-way ANOVA with Dunnett’s *post hoc* test or two-way ANOVA with Bonferroni *post h*oc test. In cases in which data were non-normally distributed, a Mann–Whitney *U* test was applied (**Figure 1**). A *P*-value of less than 0.05 was considered to be statistically significant. All statistical analyses were performed using GraphPad Prism version 5 or later.

## Results

### CK1δ and ε Are Highly Upregulated in the Lungs of IPF Patients

The distribution and level of immunoreactive CK1δ and CK1ε are altered in IPF (**Figure 1**), with each showing a striking increase in level at the cellular level (i.e., independent of the increase in tissue area per field of view). Expression is evident in multiple cell types, based on location and cell shape. Moreover, one of the biologically important substrates for CK1δ and CK1ε, Period-2 showed increased levels of expression and phosphorylation in IPF parenchymal sections, consistent with inferred increased net activity of the CK1δ/ε isoforms (**Figure 1**).

As TGF-β induces fibrosis through both the activation of fibroblasts and the induction of EMT (Leask and Abraham, 2004), we next sought to establish whether PF670462 modulated TGF-β-activated fibrogenic pathways.

### PF670462 Softens Primary Human Fibroblast Spheroids

**FIGURE 2:**
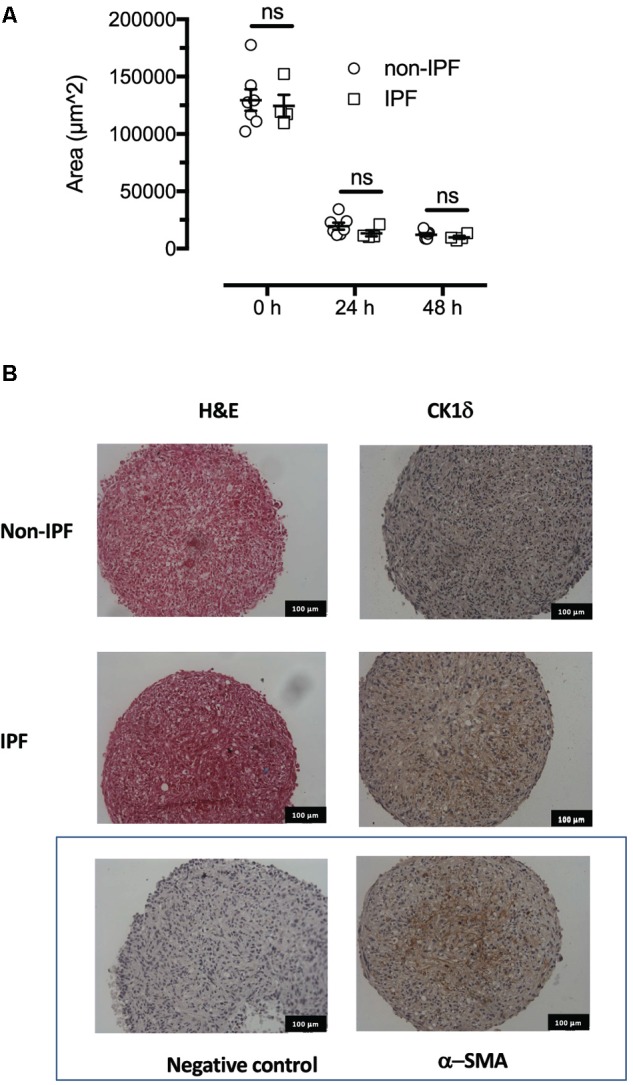
Formation and morphology of fibroblast spheroids. The spheroids remodel over the first 24 h since formation assuming a smaller and consistent volume by 48 h. **(A)** The IPF derived spheroids (*n* = 4) remodel and assume a similar volume to fibroblast spheroids from non-IPF donors (*n* = 7). **(B)** Haematoxylin and eosin staining showed similar morphology at 48 h in 5 micron sections. Immunoreactive CK1δ was detected in both non-IPF and IPF spheroids in sections representative of *n* = 7 and 4 donors, respectively, that also showed low background staining (non-immune isotype negative control) and positive staining with α-smooth muscle actin. ns, not significant.

A three-dimensional *in vitro* model to resemble fibroblast foci seen in fibrotic lesions was developed with cell spheroids formed from primary cells from IPF donors and non-diseased controls, the morphology of which is shown in **Figure 2**. Cells aggregated into spheroids and were incubated over 48 h, with most remodeling occurring during the first 24 h and minimal change in spheroid volume (measured by area of a cross-sectional plane) in the final 24 h. There was no difference in the size of spheroids from IPF or non-diseased control donors during or at the conclusion of the spheroid remodeling (**Figure 2**). Both non-diseased and IPF derived spheroids showed CK1δ immunoreactivity compared to the negative IgG isotype control. For contrast, α-smooth muscle actin was also used as a positive control, and indicated the presence of myofibroblasts within the spheroids (**Figure 2**).

**FIGURE 3:**
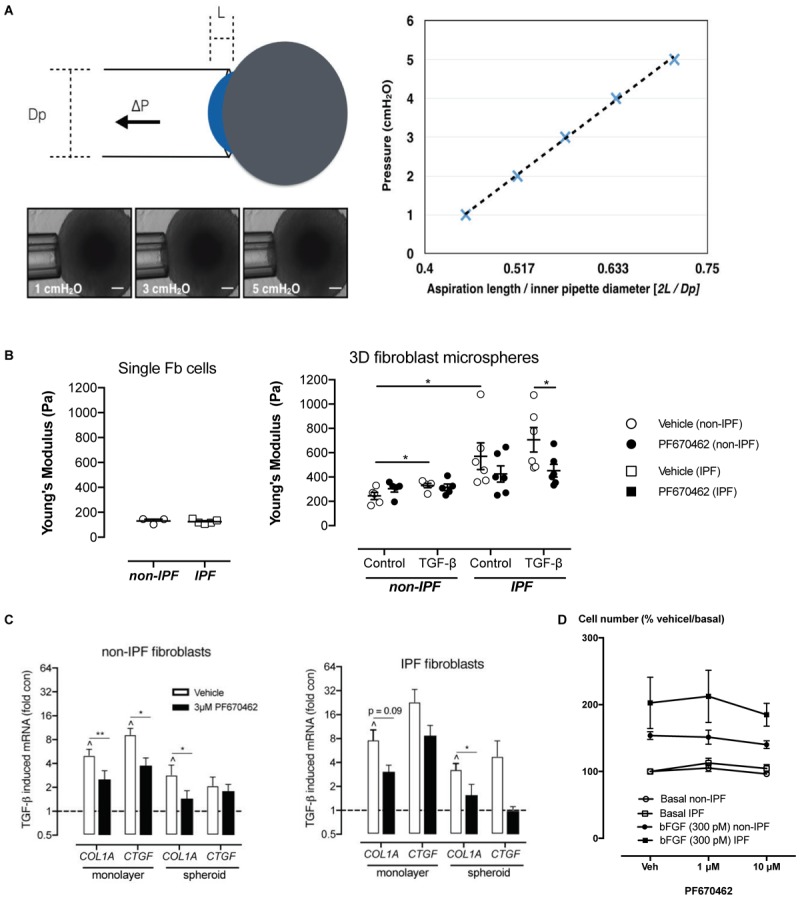
**(A)** Measurement of Young’s modulus. With increasing pressure (P) the spheroid travels a distance (L) through the micropipette barrel of inner diameter (Dp). A representative spheroid is shown with aspiration at 3 different pressures. The normalized distance (2L/Dp) is linearly related to the applied pressure and the slope of the linear regression defines the Young’s modulus. Scale bars indicate 100 μm. **(B)** Spheroid stiffness was measured by micropipette aspiration and quantified to measure the Young’s Modulus of stiffness in single cells (non-IPF, *n* = 3; IPF, *n* = 5) and in spheroids (non-IPF, *n* = 6; IPF, *n* = 6). **(C)** The effects of PF670462 on TGF-β (100 pM)-induced *COL1A* and *CTGF* mRNA expression from pFb monolayers and spheroids from IPF and non-IPF pFbs are presented as the means and SEM of (for monolayer expression: non-IPF, *n* = 3; IPF, *n* = 5) and (for spheroid expression: non-IPF, *n* = 9; IPF, *n* = 6). **(D)** Cell number expressed as a percentage of the basal cell number in the presence and absence of vehicle (Veh) or PF670462 in cells from non-IPF or IPF donors in the presence and absence of the mitogen, bFGF (*n* = 6). ^∗^*P* < 0.05, ^∗∗^*P* < 0.01 from two-way ANOVA with Bonferroni *post hoc* test or 1-way ANOVA with Dunnet’s *post hoc* test. ^∧^*P* < 0.05 from one-sample *t*-test compared to 1.0.

Fibroblasts from normal and IPF patients are known to be responsive to the stiffness of their mechanical microenvironment, with increases in contractile and proliferative phenotype observed when cells are in contact with stiffer extracellular matrix (Marinkovic et al., 2013). Stiffness can be measured by aspiration of cells and spheroids in to the barrel of a micropipette using a pressure gradient. The lesser the displacement into the barrel for a given amount of pressure the “stiffer” the material. Importantly, whilst we observed no difference in mechanical stiffness in individual cells derived from IPF or non-IPF donors, cell spheroids generated from IPF fibroblasts showed significantly higher stiffness compared to non-IPF donors (**Figure 3**), suggesting that the stiffening is an emergent property that may enhance the pro-fibrotic phenotype of these cells when culture = d as spheroids. Exposure to the fibrogen TGF-β for 48 h increased the stiffness of non-IPF spheroids (*P* < 0.05, *n* = 5), but not to the level of stiffness of IPF spheroids (**Figure 3**). Pretreatment with PF670462 (3 μM) for 30 min prior to TGF-β exposure reduced the level of stiffness in IPF spheroids (*P* < 0.05, *n* = 6) (**Figure 3**). Thus, PF670462 softens IPF fibroblast spheroids and thereby potentially interferes with the positive-feedback cycle of stiffness-induced stiffening.

### PF670462 Inhibits Fibrogenic Gene Expression in 2D and 3D Culture Systems

We used fibroblast monolayers and spheroids to examine the effect of PF670462 on TGF-β_1_ fibrogenic gene activation. Two IPF cultures and one non-diseased control culture that did not respond to TGF-β_1_ stimulation were excluded from further analysis in this and other fibroblast studies. IPF fibroblast cultures showed enhanced TGF-β induction of the genes *COL1A* and *CTGF* compared to non-IPF derived fibroblasts, whereas fibroblast spheroids showed decreased gene induction compared with fibroblast monolayers regardless of disease status of donors (**Figure 3A**). Pretreatment with PF670462 inhibited the TGF-β-induced increase in COL1A and CTGF mRNA in fibroblast monolayers and spheroids from both IPF and non-diseased control donors (**Figure 3C**). TGF-β_1_ itself showed no effect on fibroblast cell proliferation (data not shown). Basic fibroblast growth factor (bFGF)-induced mitogenesis was only marginally reduced by PF670462 at 10 μM (**Figure 3D**), suggesting that inhibition of fibroblast proliferation is not a major anti-fibrotic mechanism for PF670462.

We also sought to examine the effects of PF670462 in comparison with two clinically used anti-fibrotic agents indicated for IPF, nintedanib and pirfenidone. Non-IPF fibroblasts were incubated with nintedanib (0.3 μM), a concentration chosen for its proximity to the Cmax of ∼70 nM when taken orally at 150 mg twice daily (Ogura et al., 2015) and to avoid overt cytotoxicity noted at 3 μM. Pirfenidone was used at a concentration of 100 μM also chosen for its proximity to the reported Cmax of ∼70 μM when 801 mg is taken orally in the fasted state as a single tablet (Pan et al., 2017). The concentration of PF670462 was chosen based on our previous concentration-response analyses, since Cmax is not known, and indeed likely to be irrelevant for the efficacy of an inhalational agent. Pirfenidone (100 μM) caused a significant and pronounced reduction in TGF-β–induced Col1A mRNA levels without affecting either CTGF or αSMA expression. Nintedanib significantly reduced Col1 A and αSMA, but not CTGF. In contrast, 1 μM PF670462 reduced expression of each of these fibrogenic genes (**Table 4**).

**Table 4:** Modulation of TGF-β induced gene expression in non-IPF lung fibroblasts.

mRNA expression (% TGF-β level)			
Gene	Nintedanib 0.3 μM	Pirfenidone 30 μM	PF670462 1 μM
CTGF	70.2 ± 20.5 (10) ns	75.6 ± 20.0 (11) ns	56.5 ± 14.7 (7)^∗^
Col1A	51.9 ± 15.8 (8)^∗^	21.1 ± 5.0 (9)^∗^	33.9 ± 12.5 (5)^∗^
αSMA	64.4 ± 15.0 (10)^∗^	72.0 ± 18.2 (11) ns	71.2 ± 9.9 (7)^∗^

*Mean and SEM of (n) observations. ^∗^P < 0.05, paired Students t-test vs. level of gene expression detected in vehicle incubated TGF-β exposed cultures (100%).*

### PF670462 Inhibits Epithelial-Mesenchymal Transition of Alveolar Epithelial Cells

**FIGURE 4:**
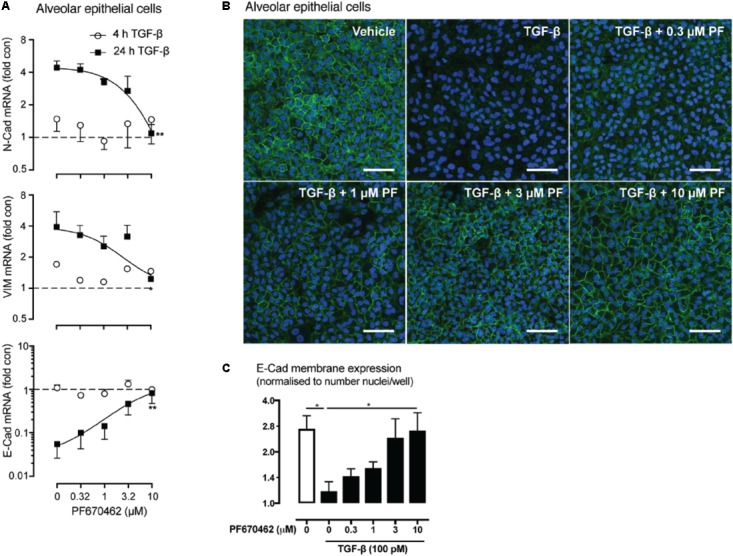
**(A)** Effect of PF670462 on EMT associated gene induction in A549 alveolar epithelial cells 4 and 24 h after TGF-β (100 pM) stimulation (*n* = 4). **(B)** Immunofluorescence of membrane *E*-cadherin expression in A549 alveolar epithelial cells. Cells were treated with TGF-β (100 pM) for 48 h with PF670462 added 30 min prior to TGF-β. Nuclei were stained with DAPI. Quantification of *n* = 4 experiments is shown in **(C)** with >50 fields analyzed per well. Data are presented as mean ± SEM from 4 independent experiments on A549 epithelial cells. ^∗^*P* < 0.05, ^∗∗^*P* < 0.01 from two-way ANOVA with Bonferroni *post hoc* test or one-way ANOVA with Dunnet’s *post hoc* test. ^∧^*P* < 0.05 from one-sample *t*-test compared to 1.0.

We investigated whether PF670462 modulates TGF-β signaling of EMT in A549 cells, measuring regulation of the EMT-associated genes *N*-Cadherin (*N*-Cad), Vimentin (Vim) and *E*-Cadherin (*E*-Cad) after 24 h of TGF-β exposure, with no change being detected at the 4 h timepoint (**Figure 4A**). These delayed gene expression changes were inhibited by PF670462 in a concentration-dependent manner, reaching a maximum effect at 10 μM (**Figure 4A**). To confirm a functional effect of PF670462 in preventing TGF-β induced EMT, *E*-cadherin was immunostained. PF670462 concentration-dependently inhibited TGF-β-induced loss of *E*-cadherin expression, reaching a maximum effect at 3–10 μM PF670462 (**Figures 4B,C**).

**FIGURE 5:**
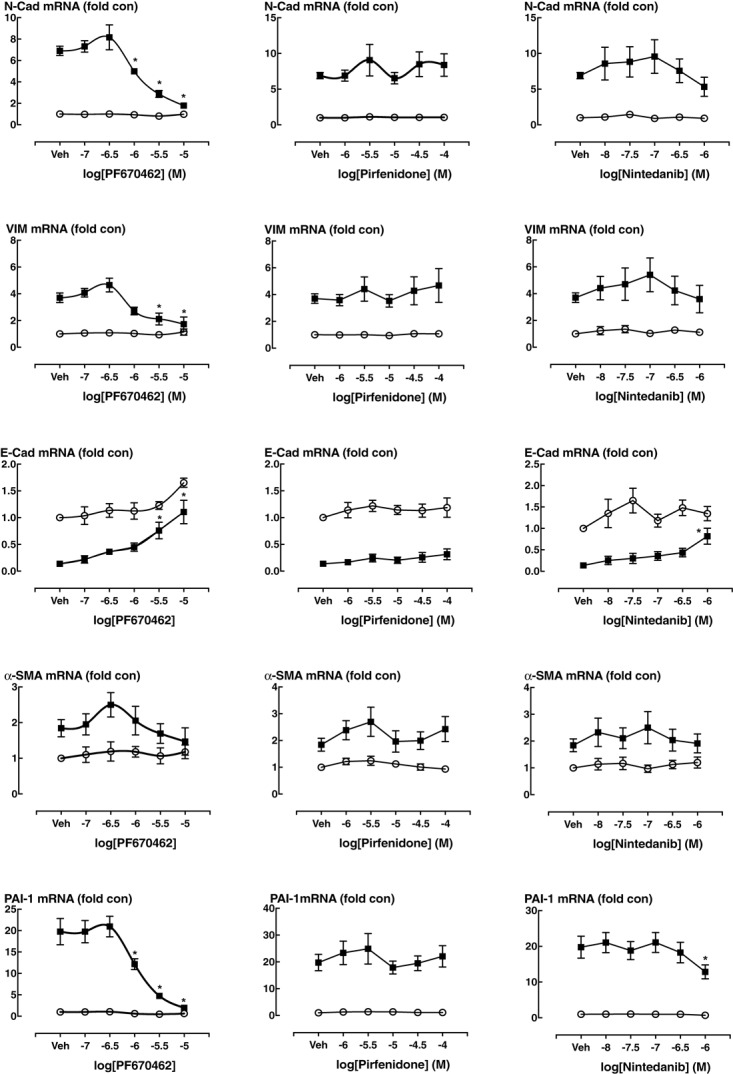
The EMT gene expression at baseline and in the presence of TGF-β (100 pM, 24 h incubation) was measured in the presence of vehicle (Veh, 0.1% DMSO) or over a range of concentrations of PF670462 (0.1 – 10 μM), Pirfenidone (1 –100 μM)) or nintedanib (10 –1000 nM) in. Data are presented as mean and SEM of *n* = 4 independent experiments and show the TGF-β induced fold increase in the expression of genes that change during EMT or in response to TGF-β in A549 cells, including *N*-cadherin (*N*-Cad), Vimentin (Vim), *E*-Cadherin (*E*-Cad), α-smooth muscle actin (a-SMA) plasminogen-activator inhibitor-1 (PAI-1). Data were analyzed by two-way ANOVA with repeated measures, followed by comparisons at individual concentrations using Bonferroni’s correction for multiple comparisons. ^∗^*P* < 0.05.

We also undertook comparative assessment of the activity of nintedanib, pirfenidone and PF670462 on markers of EMT in A549 cells (**Figure 5**). PF670462 concentration-dependently and fully prevented all of the TGF-β effects. Pirfenidone was without any significant effects up to 100 μM, whereas nintedanib showed activity on *N*-Cad, *E*-Cad and PAI-1 at the highest concentration of 1 μM.

### PF670462 Attenuates Bleomycin-Induced Fibrosis in Acute and Chronic Mouse Models

**FIGURE 6:**
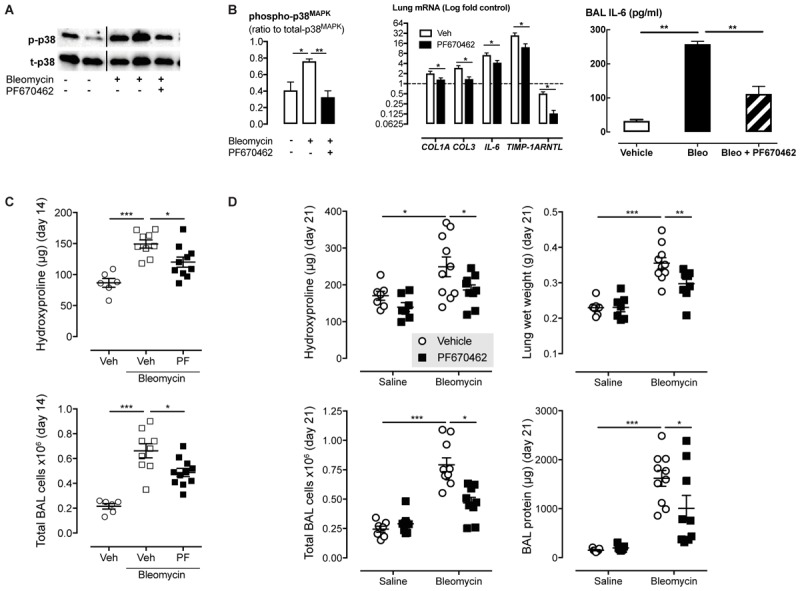
The dual CK1δ/ε inhibitor PF670462 prevents and attenuates bleomycin-induced pulmonary fibrogenesis in mice. Male C57Bl/6 mice received an intranasal dose of bleomycin or saline on day 0. PF670462 (30 mg/kg/day, i.p.). **(A,B)** p38^MAPK^ phosphorylation and fibrogenic gene expression in lungs from mice treated with PF670462 from day – 1 to 3 as measured on day 3 (*n* = 5 per group). Western blots of 2 representative mice are shown and data from all mice quantitated and tested. **(C)** Hydroxyproline content and bronchoalveolar lavage (BAL) cell number in lungs from female mice treated with PF670462 from day 3–13, as measured on day 14 (*n* = 6 sal, *n* = 12 bleo groups). **(D)** Hydroxyproline content and wet weight of lungs and BAL cell number and protein content from female mice treated with PF670462 from day 7–21 as measured on day 21 (*n* = 7 sal, *n* = 9–10 bleo groups). Data are presented as mean ± SEM. ^∗^*P* < 0.05, ^∗^*P* < 0.01, ^∗∗∗^*P* < 0.001 from one-way ANOVA with Dunnet’s *post hoc* test, or two-way ANOVA with Bonferroni *post hoc* test.

Since CK1δ and ε have been implicated in TGF-β signaling and showed greatly increased levels and activity in IPF lungs, we examined the effect of the dual CK1δ/ε inhibitor PF670462 in preventing and therapeutically treating bleomycin-induced pulmonary fibrosis in mice. We first assessed early fibrogenic responses in mice, 3 days after transnasal pulmonary bleomycin exposure. The increase in p38 MAPK phosphorylation was prevented by systemic PF670462 (30 mg/kg/day i.p. from day -1 to 3) (**Figure 6A**), a dosing regimen based on preclinical work showing that this daily i.p. dose was sufficient to cause a phase shift in the central CLOCK (Sprouse et al., 2010). This latter finding has been confirmed in a number of studies (Badura et al., 2007; Walton et al., 2009; Meng et al., 2010; Kennaway et al., 2015) and applied in others, to examine the impact of CK1δ inhibitors on drug seeking behavior (Bryant et al., 2009; Perreau-Lenz et al., 2012). Although PF670462 is known to be subject to rapid hepatic metabolism and has a quoted T1/2 of less than 30 min (Wager et al., 2014), it is evident from the cited findings that peripheral blood levels are sufficient to achieve inhibition of the central CLOCK, and are therefore sufficient to block the target in peripheral tissues, as further indicated by control of hepatic CLOCK genes (Kennaway et al., 2015). Fibrogenic gene induction was reduced (IL-6, TIMP-1) or prevented (COL-1A, COL-3) by PF670462 treatment and BAL levels of immunoreactive IL-6 (**Figure 6B**). We also demonstrate the suppression of ARNTL expression, which itself is partially suppressed by bleomycin.

To evaluate the anti-fibrotic potential of PF670462, treatment was commenced at day 3 (peak of inflammation) continuing until day 13 before *post mortem* at day 14. Lung collagen was assessed by measurement of the collagen-specific modified amino acid, hydroxyproline. PF670462 (30 mg/kg/day, i.p.) attenuated bleomycin-induced accumulation of hydroxyproline and the number of infiltrating immune cells measured in the BAL fluid (**Figure 6C**).

To further restrict the potential for a PF670462 treatment effect on inflammation, we delayed treatment until day 7 post-bleomycin, extending the period day 21 to allow sufficient time for an anti-fibrotic of this delayed treatment to be detected. Using this ‘therapeutic’ treatment regimen, bleomycin-induced increases in lung collagen (hydroxyproline), oedema (lung wet weight), BAL fluid infiltrating cells and protein were reduced by PF670462 treatment (**Figure 6D**).

**FIGURE 7:**
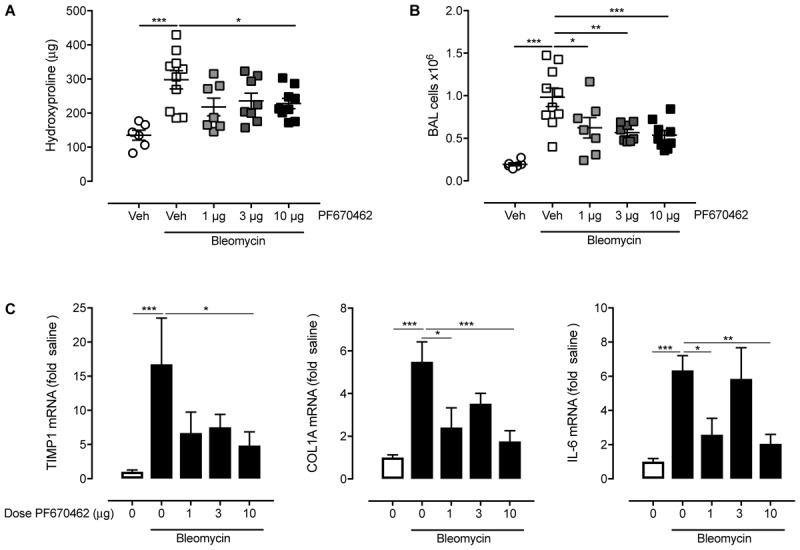
Effect of inhaled PF670462 (day 8–21) on bleomycin-induced collagen deposition and BAL cell recruitment in mouse lungs. Female C57Bl/6 mice received intranasal bleomycin or saline on day 0. PF670462 was administered on days 8–20 by the daily inhalation of aerosol of increasing concentration from 0.3 to 3 mg/mL, for a period of 15 min once daily. Collagen content (**A**; measured by assessment of lung hydroxyproline content), Bronchoalveolar lavage (BAL) cell number **(B)** and lung fibrogenic gene expression **(C)** were assessed on day 21. Data are presented as mean ± SEM (*n* = 6 sal, *n* = 9–10 bleo groups). ^∗^*P* < 0.05, ^∗∗^*P* < 0.01, ^∗∗∗^*P* < 0.001 from one-way ANOVA with Dunnet’s *post hoc* test.

In a separate study in female mice, aerosolised PF670462 (0.3–3.0 mg/ml, 15 min/once daily) corresponding to estimated deposited doses of ∼1 – 10 μg/day (Oldham and Robinson, 2007) from day 8–20, also reduced hydroxyproline content, BALF cell influx, and fibrogenic gene expression at day 21 (**Figure 7**), albeit that the effects of PF670462 did not show dose-related effects on these measures over this range of exposures.

## Discussion

In this study, we have demonstrated striking up-regulation of CK1δ and ε in the lungs of IPF patients. Furthermore, in 4 separate studies we show that the dual CK1δ/ε inhibitor PF670462 exhibits anti-fibrotic effects in early and later phases of the fibrogenic response to bleomycin. In late 2014 the first two drugs were approved by the Food and Drug Administration (United States) for the treatment of IPF. Hitherto, treatment relied on azathioprine, systemic glucocorticoids and N-acetyl cysteine, which have since been found to shorten survival (Idiopathic Pulmonary Fibrosis Clinical Research Network et al., 2012). Treatment with nintedanib over extended periods reduces the decline in lung function compared to placebo (Richeldi et al., 2014). Nintedanib is a tyrosine kinase inhibitor that inhibits the ATP binding site of a host of tyrosine kinase receptors, including those for PDGF, FGF and VEGF. Pirfenidone, on the other hand, has anti-inflammatory, anti-fibrotic and anti-oxidant effects through an as yet undefined receptor that involves inhibition of TGF-β production, reduced fibroblast activation, and a decrease in extracellular matrix production (King et al., 2014; Selvaggio and Noble, 2016). Importantly, as both of these orally administered drugs have significant systemic adverse-effects, there remains great scope for further therapeutic gains from new agents, delivered by inhalation to minimize systemic adverse effects.

The bleomycin mouse model of fibrosis has been criticized for poor predictive value (Mercer et al., 2015). However, the use of a “therapeutic” drug dosing regimen, in which drugs are administered after the inflammatory phase of the model (as in the current study), is thought to be more predictive of clinical outcome (Moeller et al., 2008). Indeed, the impact on collagen deposition of PF670462 treatment (either systemically 30 mg/kg/day or by aerosol 1 μg/day) once daily in the bleomycin mouse model is comparable with reports of the twice daily impact of dosing of 400 mg/kg/day of pirfenidone (Kakugawa et al., 2004). Pirfenidone is known to modulate TGF-β pathways, but there is no discrete molecular target against which the drug class can be further optimized. PF670462 may be more suitable than pirfenidone as a TGF-β modulator, as its potency enables inhalational delivery, thereby limiting the systemic adverse effects. Head-to-head studies in the murine model of fibrogenesis may add weight to the existing contrasts on *in vitro* fibrogenesis models using cultured human cells.

Two features of PF670462 support its suitability as a TGF-β modulator. Firstly, PF670462 shows selectivity for inhibition of CK1δ/ε –dependent as opposed to other actions of TGF-β, as expected from its selectivity for CK1δ over the TGF-β receptor kinase, ALK5. This selectivity avoids compromising TGF-β dependent Treg cell populations, the loss of which are implicated in gut autoimmune colitis resulting from global TGF-β inhibition, as discussed in our recent review on safety of TGF-β modulation (LaChapelle et al., 2018). Secondly, the greatly diminished systemic exposure achieved by inhalational usage reduces the risk of cardiac or gut autoimmune defects, and minimizes the risk of disruption to central circadian rhythm.

Studies on the mechanism of PF670462 suggest suppression of EMT, in addition to regulatory effects on lung fibroblasts, presenting a pharmacological profile that is distinct to nintedanib, which reduces (myo)fibroblast activity, with limited effect on EMT (Wollin et al., 2015). We acknowledge limitations in the utility of A549 cells to represent native alveolar epithelial cells. Nevertheless, A549 cells recapitulate the hallmarks of EMT that are observed in *in vivo* studies in reporter mice and human IPF patients (Kasai et al., 2005; Kim et al., 2006), and our data unequivocally indicate modulation of TGF-β effects.

As TGF-β is a very well validated key target in fibrosis, there is considerable interest in investigating selective targeting strategies. Recent efforts have identified that targeting the α_v_β_6_ integrin, that is strongly upregulated at fibrogenic sites, reduces fibrosis-related TGF-β activation whilst avoiding TGF-β suppression in healthy tissue (Horan et al., 2008; Puthawala et al., 2008). This strategy seems promising. BG00011 (aka STX-100), a humanized monoclonal antibody targeting the α_v_β_6_ integrin, is currently in phase 2 clinical trials (NCT01371305). PF670462, as a small molecule, has many advantages over a monoclonal antibody, including ease and route of administration, and lower cost of goods. Furthermore, our observation of local efficacy following inhalation in the bleomycin-mouse model enables minimization of the systemic adverse-effects of targeting TGF-β pathways.

Our results suggest that PF670462 or similar CK1δ/ε inhibitors could provide a promising new approach for the treatment of pulmonary fibrosis. The beneficial actions of PF670462 in highly relevant 3D spheroid models using both non-diseased and IPF-derived fibroblasts, considered together with the impact of the ‘therapeutic’ dosing regimen in the mouse studies, support arguments for further preclinical development of PF670462 or other agents targeting CK1δ/ε. Minimization of the systemic adverse-effects of targeting TGF-β pathways will be important in the development of this agent. The potential impact of the inevitable off-target effects of small molecule kinase inhibitors CK1δ/ε will be minimized by their inhalational use. There are predictable on-target adverse effects of systemic CK1δ/ε inhibitors, including modulation of circadian rhythm (Badura et al., 2007; Meng et al., 2010). Inhalational use of CK1δ/ε inhibitors will minimize unwanted systemic actions, as will the low oral availability of PF670462 that is subject to rapid first-pass hepatic metabolism (Wager et al., 2014). Although we propose that CK1δ/ε is playing a role in selected fibrogenic TGF-β signaling, recent findings demonstrating the *Clock*^Δ*19*^ mice that lack circadian variation in anti-oxidant enzymes, show greater fibrogenic response to bleomycin (Pekovic-Vaughan et al., 2014), raises the potential for additional benefits of CK1δ/ε inhibitors to those of modulating downstream TGF-β signaling.

## Conclusion

Our evidence indicates that both CK1δ and ε are highly up-regulated in IPF lungs and their inhibition with the dual kinase inhibitor PF670462 has broad anti-fibrotic efficacy in pre-clinical models. We therefore suggest that PF670462 or other inhibitors of CK1δ/ε are promising candidates for development as inhalational anti-fibrotic agents.

## Datasets Are Available on Request

The raw data supporting the conclusions of this manuscript will be made available by the authors, without undue reservation, to any qualified researcher.

## Author Contributions

CK, SL, DP, ML, TH, MS, YT, FJ, YX, and BG acquired and analyzed the data. CK, SL, PL, and AS interpreted the data. JJ and GW provided the human lung specimens from IPF and non-IPF donors from which TH generated primary human fibroblast cell cultures. AS conceived the study. CK and AS wrote and AS revised the manuscript. All authors edited and approved submission of the manuscript.

## Conflict of Interest Statement

AS, CK, and TH are co-inventors on a patent protecting the use and formulation of inhaled PF670462 in respiratory disease. The remaining authors declare that the research was conducted in the absence of any commercial or financial relationships that could be construed as a potential conflict of interest.
